# Bacteriomimetic Liposomes Improve Antibiotic Activity of a Novel Energy-Coupling Factor Transporter Inhibitor

**DOI:** 10.3390/pharmaceutics14010004

**Published:** 2021-12-21

**Authors:** Menka Drost, Eleonora Diamanti, Kathrin Fuhrmann, Adriely Goes, Atanaz Shams, Jörg Haupenthal, Marcus Koch, Anna K. H. Hirsch, Gregor Fuhrmann

**Affiliations:** 1 Helmholtz Institute for Pharmaceutical Research Saarland (HIPS), Helmholtz Centre for Infection Research (HZI), Campus E8.1, 66123 Saarbrücken, Germany; menka.drost@fau.de (M.D.); Eleonora.Diamanti@helmholtz-hips.de (E.D.); Kathrin.Fuhrmann@helmholtz-hips.de (K.F.); Adriely.Goes@helmholtz-hips.de (A.G.); Atanaz.Shams@helmholtz-hips.de (A.S.); Joerg.Haupenthal@helmholtz-hips.de (J.H.); Anna.Hirsch@helmholtz-hips.de (A.K.H.H.); 2 Department of Biology, Pharmaceutical Biology, Friedrich-Alexander-University Erlangen-Nürnberg (FAU), Staudtstr. 5, 91058 Erlangen, Germany; 3 Helmholtz International Lab for Anti-Infectives, Campus E8.1, 66123 Saarbrücken, Germany; 4 INM-Leibniz-Institut für Neue Materialien, Campus D2.2, 66123 Saarbrücken, Germany; Marcus.Koch@leibniz-inm.de; 5 Department of Pharmacy, Saarland University, Campus C1.7, 66123 Saarbrücken, Germany

**Keywords:** liposomes, nanoantibiotics, energy-coupling factor (ECF) transporters, bacteriomimetic, *Bacillus subtilis*, cardiolipin, antibiotic resistance

## Abstract

Liposomes have been studied for decades as nanoparticulate drug delivery systems for cytostatics, and more recently, for antibiotics. Such nanoantibiotics show improved antibacterial efficacy compared to the free drug and can be effective despite bacterial recalcitrance. In this work, we present a loading method of bacteriomimetic liposomes for a novel, hydrophobic compound (**HIPS5031**) inhibiting energy-coupling factor transporters (ECF transporters), an underexplored antimicrobial target. The liposomes were composed of DOPG (18:1 (Δ9-*cis*) phosphatidylglycerol) and CL (cardiolipin), resembling the cell membrane of Gram-positive *Staphylococcus aureus* and *Streptococcus pneumoniae*, and enriched with cholesterol (Chol). The size and polydispersity of the DOPG/CL/± Chol liposomes remained stable over 8 weeks when stored at 4 °C. Loading of the ECF transporter inhibitor was achieved by thin film hydration and led to a high encapsulation efficiency of 33.19% ± 9.5% into the DOPG/CL/Chol liposomes compared to the phosphatidylcholine liposomes (DMPC/DPPC). Bacterial growth inhibition assays on the model organism *Bacillus subtilis* revealed liposomal **HIPS5031** as superior to the free drug, showing a 3.5-fold reduction in CFU/mL at a concentration of 9.64 µM. Liposomal **HIPS5031** was also shown to reduce *B. subtilis* biofilm. Our findings present an explorative basis for bacteriomimetic liposomes as a strategy against drug-resistant pathogens by surpassing the drug-formulation barriers of innovative, yet unfavorably hydrophobic, antibiotics.

## 1. Introduction

Following decades of successful antibiotic therapy, well-established drugs are now suffering from an accelerating loss of efficacy due to the rise of resistant pathogens [1]. Increasing morbidity and mortality [2] associated with drug-resistant pathogens such as Gram-positive *Enterococcus faecium* and *Staphylococcus aureus* [3,4,5] hence set the urgent demand for alternate antibiotic strategies. The exploration and development of novel antimicrobial targets and effective drugs, respectively, continue to be the most potent force against difficult-to-treat bacterial infections. A promising antibiotic approach is the inhibition of uptake transporters for essential micronutrients in prokaryotes, in particular when these are lacking respective biosynthetic pathways. Energy-coupling factor transporters (ECF transporters) are a subclass of ATP-binding cassette (ABC) transporters that are absent in humans but present in approximately 50% of prokaryotic species [6,7]. ECF transporters mediate the uptake of B-type vitamins (e.g., folate, thiamine, and biotin) and transition metal ions. They are composed of two cytoplasmic ATPase components (EcfA and EcfA‘) and two integral membrane proteins: EcfT (T-component) and EcfS (S-component). EcfT connects the two nucleotide-binding domains with the S-component, which is responsible for substrate binding. EcfT, together with the nucleotide-binding subunits, forms a ternary complex named the energizing or ECF-module. ECF transporters are classified into two groups, group I and -II. In the ECF transporters of group I, substrate binding proteins associate with a specific ECF-module, while in the ECF transporters of group II, several S-components can interact with a common ECF-module [7,8]. In auxotrophic prokaryotes, the uptake of certain micronutrients from the environment is crucial for survival, growth, and virulence. *S. aureus* expresses S-components of group II for riboflavin, a thiamin precursor, and biotin, but the pathogen is also equipped with respective biosynthetic pathways. *Enterococcus faecium*, unable to resort to the de novo synthesis of folic acid, must acquire folate via transport proteins [6].

**Figure 1:**
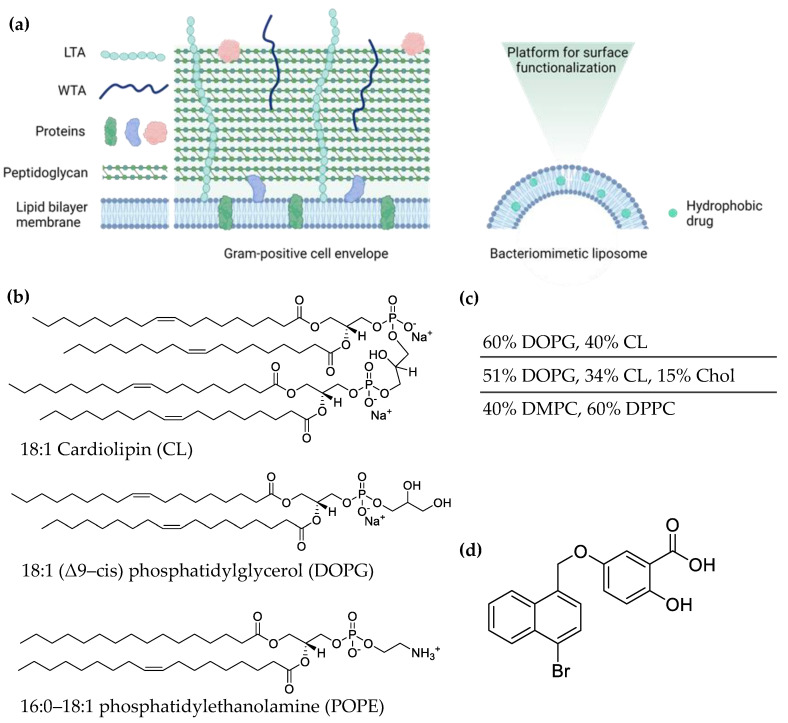
(**a**) Scheme of the Gram-positive cell envelope and bacteriomimetic DOPG/CL liposomes. LTA, lipoteichoic acid; WTA, wall teichoic acid. (**b**) Phospholipids present in the cell membrane of Gram-positive *S. aureus* and *S. pneumoniae*. (**c**) Lipid composition of liposomes prepared in this work. Phosphatidylcholine (PC) is the main phospholipid of eukaryotic cells. (**d**) Structure of **HIPS5031**, an inhibitor of Energy-coupling factor (ECF) transporters that are responsible for micronutrient uptake in prokaryotes. Figure 1a was created with BioRender.com.

Beyond the discovery of novel antimicrobial targets and drugs, drug formulation and delivery contribute significantly to antibiotic efficacy in the context of bacterial tolerance and resistance. A galenic key concern is drug solubility. Sufficient bioavailability [9] is indispensable for efficient and safe antibiotic therapy, as the requirement for high antibiotic dosage, resulting from poor solubility, enhances the resistance formation of pathogens [10] and commensals [11]. In this work, we prepared a liposomal formulation of a novel compound related to previously reported inhibitors of ECF-transporters [12], **HIPS5031** (Figure 1d, comprehensive information on the synthesis and characterization of **HIPS5031** is given in the SI). Liposomes are a well-recognized strategy for improving the solubility of lipophilic compounds, which is of increasing significance since the majority of newly developed drugs are considered to be poorly soluble [13]. Similar to biological membranes, the main constituents of liposomes are amphiphilic lipids composed of polar headgroups and hydrophobic acyl chains [14]. Thus, incorporation of the highly hydrophobic compound **HIPS5031** (cLogP~6, calculated with ChemDraw, Figure 1d) into the lipid bilayer of liposomes is a feasible approach to improve its solubility and, potentially, its antibacterial activity. As nanoparticulate drug delivery systems, liposomes offer a plethora of biopharmaceutical advantages and can be used as a technological platform for controlling the site and mode of drug release [15,16,17]. When used as nanoantibiotics [18], the potential of liposomes for selective and controlled drug delivery, accumulation at infection sites, local increases in antibiotic concentration, and activity against intracellular pathogens is of particular interest, as these effects cumulate in both enhanced antibiotic efficacy and a reduction in side effects [15,19,20].

Liposomes are also capable of surpassing strong permeation barriers that are specific to bacterial cells, mainly the cell wall and the complex extracellular matrix of biofilms [21]. The ability of liposomes to interact with cells or extracellular substances is directly influenced by their lipid composition [22,23]. While mammalian cell membranes are mainly composed of phosphatidylcholines (PC) and phosphatidylethanolamines (PE), bacterial membranes consist in varying ratios of PE, phosphatidylglycerols (PG), and cardiolipin (CL) [24,25]. Cardiolipin, only found in prokaryotic plasma membranes (≤10%) and in the inner mitochondrial membrane of eukaryotes, presents an extraordinary, dimeric phospholipid with two phosphatidic acid moieties linked together by a central glycerol group. The small and two-fold negatively charged (pH 7.4) polar head-group and the extensive hydrophobic region of the cardiolipin molecule lead to a condensation of bilayers and affect the shape and size of liposomes [26,27,28]. Gram-negative bacteria are characterized by a high membrane content of PE with wild-type *E. coli* reaching up to 80%. The PE-content in membranes of Gram-positive bacteria, on the other hand, is lower, or PE is absent. *Bacillus subtilis* membranes are composed of PE/PG/CL in a ratio of 15/80/5% [29], while *S. aureus* and *S. pneumoniae* membranes are composed of only PG and CL (58% PG, 42% CL in *S. aureus*) (Figure 1) [24]. In this work, pure PC liposomes resembling the zwitterionic outer monolayer of eukaryotic plasma membranes [24] were compared to negatively charged bacteriomimetic liposomes [30]. Membrane fluidity and permeability in cells is further regulated by planar, polycyclic hydrocarbons, namely cholesterol in the plasma membranes of mammalian cells and hopanoids in some Gram-negative and Gram-positive bacteria. Both hydrocarbons are known to condense and thicken membranes by intercalation into the bilayer, which leads to a decrease in membrane fluidity and permeability [31]. Thus, bacteriomimetic lipid compositions were enriched with cholesterol (15%) in order to assess the influence of membrane rigidity on encapsulation efficiency and antibacterial activity.

With the aim of promoting new antimicrobial strategies against critical pathogens, liposomes resembling the lipid composition of *S. aureus* and *S. pneumoniae* were employed as a delivery system for a novel antibiotic lead compound, which was shown to inhibit the ECF-mediated uptake of folate in *Lactobacillus casei* (*L. casei*). For antimicrobial effect assays, *B. subtilis* was used as an easily culturable Gram-positive model organism, being generally recognized as non-pathogenic and the most comprehensively studied biofilm-producing species of the Firmicutes phylum [32,33]. This article reports on an efficient thin-film hydration based loading strategy for **HIPS5031**-DOPG/CL/Chol liposomes, their physicochemical characterization, and their effects against *B. subtilis* and *B. subtilis* biofilm.

By combining an innovative, yet unfavorably hydrophobic, antibiotic lead compound with a solubility-increasing nanoparticulate drug delivery system, we present a first approach towards a potential galenic formulation of an ECF-transporter inhibitor and thereby a new strategy against difficult-to-treat bacterial infections.

## 2. Materials and Methods

### 2.1. Liposome Preparation and Drug Encapsulation

Liposomes were prepared by thin film hydration. In brief, lipids (DMPC, DPPC, DOPG, cardiolipin) and cholesterol (all Avanti Polar Lipids, Alabaster, AL, US) were dissolved in chloroform and mixed to obtain different lipid compositions containing 5 mM (DMPC/DPPC and DOPG/CL) or 4.25 mM lipids (DOPG/CL/Chol). The molar ratios were 40% DMPC/60% DPPC and 60% DOPG/40% CL (51%/34% + 15% cholesterol). The open vials were left under the exhaust fume overnight to let the chloroform evaporate. The resulting lipid films were stored at −20 °C. Lipid films were rehydrated with 1 mL sterile phosphate-buffered saline (PBS, Thermo Fischer, Waltham, MA, USA) for one hour on a heating plate (42 °C for DMPC/DPPC, 72 °C for DOPG/CL ± cholesterol). Vials were sporadically vortexed to support vesicle formation. Following rehydration, the liposome dispersion was transferred into a Hamilton syringe and liposomes were extruded on a pre-heated (42 °C/72 °C) mini extruder (Avanti Polar Lipids, Alabaster, AL, USA) through a polycarbonate membrane with a pore size of 0.2 µm (Sigma Aldrich, Taufkirchen, Germany). The liposome stock solution was then purified by size-exclusion chromatography (SEC) on a Sepharose CL-2B column (GE Healthcare Life Science, Uppsala, Sweden) with PBS, and fractions were collected for further experiments.

Loading of **HIPS5031** was achieved by either rehydrating the dry lipid films with 50 µL of a 20 mg/mL DMSO-solution of **HIPS5031** in 950 µL sterile PBS or by incorporating the drug into lipid films and rehydration with sterile PBS. **HIPS5031** was incorporated into the lipid films by dissolving 1 mg of the drug together with the lipids in absolute ethanol (VWR International, Radnor, PA, USA) and subsequent removal of the organic solvent by placing the open vials on a heating plate (>78.32 °C, ~80 °C) for several hours.

### 2.2. Characterization of Liposomes

The hydrodynamic diameter of blank and loaded liposomes, their size distribution, and zeta potential were measured in sterile PBS with a Zetasizer Nano (Malvern Panalytical, Malvern, UK) by dynamic and electrophoretic light scattering. Measurements were performed in triplicate. The particle concentration of liposomes in PBS (stated as particles per mL (ppmL)) was determined by nanoparticle-tracking analysis (NTA) on a Nanosight instrument (Malvern Panalytical, Malvern, UK). Records obtained with a CCD camera with a tracking time of 3 min and at a camera level of 5 and detection threshold of 15 were analyzed by the NTA 3.3 software.

### 2.3. HPLC-MS

For quantification of **HIPS5031**, mass spectrometry analysis was performed on a SpectraSystems-MSQ LCMS system (Thermo Fischer, Dreieich, Germany) using a Hypersil C18 Gold column (150 × 3 mm, 5 µm). A quantity of 10 µL of the sample was separated at a flowrate of 700 µL/min, starting with 70% H_2_O (0.1% formic acid) and 30% acetonitrile (0.1% formic acid) and linearly increasing to 95% over a period of 12 min. UV spectra were recorded at 254 nm. Quantification was performed by generating a calibration function with 5 dilutions after integrating the area under the peak at a retention time of 4.8 min. Calibration standards were prepared in DMSO and to all measured samples, and 10% acetonitrile was added in order to disrupt the lipid bilayer and release **HIPS5031**.

### 2.4. Calculation of Encapsulation Efficiency (EE)

Calculation of the encapsulation efficiency (EE) was based on HPLC-MS derived drug concentrations within purified SEC-fractions and liposomal stock solutions after extrusion. EE represents the ratio between encapsulated and total drug concentrations (Equation (1)).

(1) $$\mathrm{EE}=\frac{c_{e}}{c_{total}}\;*100\;\left(\%\right)$$

Encapsulated concentrations are stated as the sum of drug content of those three SEC-fractions that showed the highest particle numbers as determined by NTA (*c_e_*). For quantification of the total concentration, liposomal dispersions were measured directly after extrusion (*c_total_*) in order to adjust for drug loss from the loading concentration (~1 mg) due to precipitation or adsorption.

### 2.5. Cryo-TEM

The morphology of loaded liposomes was explored by cryogenic transmission electron microscopy (*cryo*-TEM). A quantity of 3 µL of the sample was dropped onto a holey carbon grid (S147-4, Plano, Wetzlar, Germany) and blotted for 2 s before plunging it into liquid ethane (T = −165 °C) with a Gatan CP3 cryo-plunger (Pleasanton, CA, USA). The samples were then transferred to a *cryo*-TEM sample holder (Gatan 914, Pleasanton, CA, USA) under liquid nitrogen and analyzed at T = −173 °C by low-dose TEM bright field imaging at 200 kV accelerating voltage (JEM-2100 LaB_6_, JEOL, Tokyo, Japan). Images were obtained with a Gatan Orius SC1000 CCD camera with binning at 2 and 4 s imaging time at a resolution of 1024 × 1024 pixels.

### 2.6. ECF Transporter Inhibition Assay

The inhibitory effect of free and liposomal **HIPS5031** on ECF-transporter-mediated folate uptake in the Gram-positive model organism *L. casei* was studied in a cell-based transport assay as recently described elsewhere [34]. A description of the method is provided in the SI.

### 2.7. Bacterial Culture, Bacterial Growth Assays and Viability Assay

Photometric determination of the effects of free and liposomal **HIPS5031** and pure liposomes on *B. subtilis* growth is described in the SI.

For bacterial enumeration after antibiotic treatment, *B. subtilis* was grown in Lennox Lysogenic broth, LB (Sigma Aldrich, Taufkirchen, Germany), at 37 °C and 180 rpm for 16 h. *B. subtilis* cultures were diluted to an OD_600_ of 0.06 (~10^8^ CFU/mL) [35], and assays were performed with a final inoculum size of 5 × 10^5^ CFU/mL. The bacterial suspension was added to serial dilutions of test and control samples (free and liposomal **HIPS5031**, pure DOPG/CL/Chol liposomes and PBS, respectively) in 96-well plates. Plates were incubated for 16 h at 37 °C, 5% CO_2_, and 50 rpm, and samples were then diluted by a factor 1 × 10^6^, of which aliquots of 20 µL were transferred onto LB-Agar plates (*n* = 3). After incubation overnight at 37 °C, colony-forming units (CFU) were counted and normalized to the PBS-control.

For the viability assay, *B. subtilis* cultures were diluted to an OD_600_ of 0.06. A final inoculum size of 1.5 × 10^7^ CFU/mL was mixed with serially diluted test and control samples (**HIPS5031**-loaded DOPG/CL/Chol liposomes, **HIPS5031**, gentamicin (gentamicin sulfate, 674 IU/mg, Serva) and PBS) and 10 vol% resazurin (Invitrogen™ alamarBlue™ HS Cell Viability Reagent, Thermo Fisher Scientific, A50100) in 96-well plates. Plates were incubated statically at 37 °C and 5% CO_2_ for 4 h, and fluorescence intensity was subsequentially measured with a microplate reader (Infinite M200 Pro, Tecan Group Ltd., Männedorf, Switzerland). The measurement parameters were bottom reading and multiple reads per well (4 × 4, circle) at an excitation wavelength of 560 nm, an emission wavelength of 590 nm, a manual gain of 100, and 25 flashes. Since resazurin is non-toxic to cells, CFUs were determined after the fluorescence measurement according to the protocol described above (dilution by a factor 1 × 10^5^). Viability assays and CFU assays conducted after the fluorescence measurement were performed as biological triplicates (*n* = 9).

### 2.8. B. subtilis *168* Biofilm for SEM & Fluorescence Imaging

Biofilm experiments were adapted from the recent work published by our group [36]. For SEM imaging, biofilm was grown on sterile glass coverslips (round, 12 mm) placed inside the wells of a 24 well plate. An overnight culture of *B. subtilis* 168 grown in Lennox Lysogenic broth, LB (Sigma Aldrich, Taufkirchen, Germany), at 37 °C, 5% CO_2_, and 180 rpm was diluted in biofilm-growth-stimulating MSgg medium [37] to an OD_600_ of ~0.1 (1 × 10^6^ CFU/mL). Then, 300 µL of the bacterial suspension was transferred to the wells and incubated statically at 37 °C and 5% CO_2_. After 72 h, the supernatant was removed, and wells were carefully washed with PBS. Quantities of 250 µL of **HIPS5031**-loaded DOPG/CL/Chol liposomes (375 µM, 1.5 × 10^13^ ppmL), **HIPS5031** (375 µM), gentamicin (375 µM), and LB medium were added onto the glass coverslips. The coverslips were incubated for 24 h at 37 °C with 5% CO_2_. After treatment, the coverslips were serially dehydrated with ethanol (addition of 30, 50, 70, 80, 90, 100% ethanol for 10 min each) and subsequentially incubated with 300 µL of hexamethyldisilazane (HMDS) for 20 min. HMDS was removed and the coverslips were dried overnight under a fume hood, mounted onto carbon tape on sample holders, and gold-sputtered (Quorum Q150R ES). Images were obtained with a Zeiss EVO MA15 LaB6 scanning electron microscope at an accelerating voltage of 20 kV.

For confocal laser scanning microscopy, *B. subtilis* 168 biofilm was grown in MSgg medium in a black 96-well plate with a transparent bottom (Greiner Bio-One, 655090). An overnight culture grown in LB medium was diluted to an OD_600_ of 0.3 in MSgg medium (3.3 × 10^6^ CFU/mL) and 200 µL of the bacterial suspension was added to each well. The plate was incubated statically at 37 °C and 5% CO_2_ for 96 h. After removal of the supernatant, wells were washed with PBS and 100 µL of **HIPS5031**-loaded DOPG/CL/Chol liposomes (1.2 × 10^13^ ppmL), **HIPS5031**, gentamicin, and LB medium was added (all drug concentrations were adjusted to 2.95 µM). After incubation at 37 °C and 5% CO_2_ for 20 h, the biofilm was stained by adding 100 µL of PBS containing 1.5 µL SYTO9 and 1.5 µL propidium iodide per mL to each well followed by incubation for 15 min at 37 °C. Subsequently, 100 µL of PBS containing 20 µg/mL Hoechst 33,342 was added, followed by incubation for 15 min at 37 °C. The biofilm was then fixed in 4% paraformaldehyde for 30 min at 37 °C and the fluorescence of the SYTO9 stained nucleic acid was examined with a microplate reader (Infinite M200 Pro, Tecan Group Ltd., Männedorf, Switzerland; top reading and multiple reads per well (4 × 4, circle), 485/530 nm, manual gain of 100 and 25 flashes). The wells were then imaged with a confocal laser scanning microscope (Leica TCS SP8, Leica Microsystems, Wetzlar, Germany) and processed with LAS X software (LAS X 1.8.013370, Leica Microsystems).

### 2.9. Statistical Analysis

Data are displayed as mean ± standard deviation. The number of independent experiments is stated as *n*. Treatment with free or liposomal **HIPS5031** was compared to blank liposomes and a control, and statistical analysis was performed by one-way analysis of significance (ANOVA) or multiple t-tests (alamarBlue-based viability assay and SYTO9 fluorescence) and the Holm–Sidak test was used as a post hoc test. Significant *p*-values are stated as ** *p* < 0.01 and *** *p* < 0.001.

## 3. Results & Discussion

### 3.1. Characterization of Bacteriomimetic Liposomes

**Figure 2:**
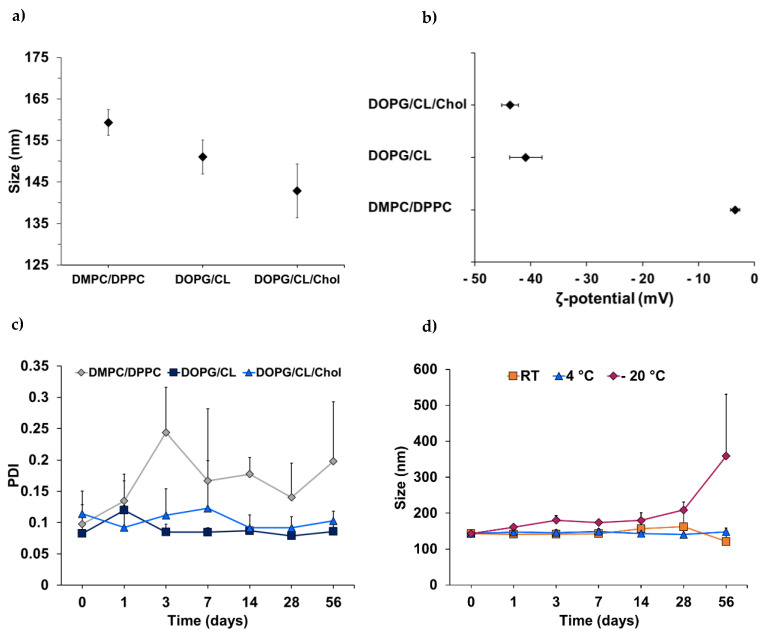
Stability assessment of bacteriomimetic liposomes: (**a**) average hydrodynamic diameter (nm) of liposomes in dispersion (PBS) as determined by DLS (Zetasizer Nano, Malvern, UK); (**b**) zeta potential (mV) of liposomes, measured in PBS; (**c**) polydispersity indices of bacteriomimetic and control (PC) liposomes stored at 4 °C over 8 weeks; (**d**) size development of DOPG/CL/Chol liposomes stored at room temperature (RT), 4 °C, and −20 °C for 8 weeks.

Liposomes were prepared and purified, and NTA was employed to determine the fraction with the highest particle concentration, which was then chosen for the testing of liposomal stability under different storage conditions (24 °C (room temperature, RT), 4 °C, and −20 °C). Stability was examined based on the zeta potential, size, and size distribution, which were determined by electrophoretic and dynamic light scattering (DLS). In accordance with the electrical neutrality of zwitterionic phosphatidylcholine [38], the DMPC/DPPC liposomes exhibited a low zeta potential of −3.5 ± 0.8 mV (Figure 2b), which is regarded as disadvantageous in terms of fusion or aggregation [39]. In contrast, the zeta potential of the DOPG/CL liposomes (±cholesterol) was highly negative and beyond the critical range of ±30 mV [40], with values of 40.9 ± 2.9 mV for the pure lipid formulation and 43.7 ± 1.5 mV for the cholesterol-enriched variant.

The impact of the surface charge and electrostatic repulsion of the particles in dispersion on their stability is reflected by changes of the polydispersity index (PDI) with high values indicating non-uniform size distribution. For drug delivery via liposomes, the particle dispersion is considered sufficiently homogeneous when PDI values are ≤0.3 [41]. When stored at 4 °C, the almost-neutral PC liposomes showed a distinct increase in PDI, starting from 0.098 ± 0.031 and rising to 0.198 ± 0.095 over the course of 8 weeks compared to the bacteriomimetic liposomes (Figure 2c). After 8 weeks of storage at 4 °C, the DOPG/CL and DOPG/CL/Chol liposomes retained low polydispersity with values of 0.086 ± 0.018 and 0.103 ± 0.016, respectively. This might likely be attributed to the electrostatic repulsion of liposomes composed of the negatively charged phospholipids PG and cardiolipin [27].

Following their superior encapsulation efficiency, cholesterol-containing DOPG/CL liposomes were compared regarding their stability when subjected to three different storage conditions (RT, 4 °C, and −20 °C). Storage at 4 °C was found to be optimal, as the hydrodynamic diameter of the liposomes fluctuated only marginally between 140 and 150 nm (Figure 2d). Freezing of the vesicles in PBS has led to a continuous increase in size, which was particularly pronounced after 28 days. These results are consistent with literature recommending storage at 4 °C [42,43], taking into account that phospholipids are prone to oxidation and hydrolysis [44], two processes amplified by elevated temperature.

Due to its bilayer-rigidifying properties, cholesterol is often added to lipid-mixtures of liposomal drug delivery systems to prevent instability and premature drug leakage [45,46]. Here, the addition of 15 mol% cholesterol to the bacteriomimetic lipid composition did not substantially enhance the storage stability of the DOPG/CL liposomes at 4 °C. This might be partially attributed to the low cholesterol content compared to the descriptions in the literature, which recommend 30 mol% [45]. However, the primary reason for the advantageous storage behavior of the bacteriomimetic liposomes, beyond electrostatic repulsion, could likely be the presence of phosphatidylglycerol and cardiolipin. Unlike the fully saturated and relatively short-chained phospholipids DMPC (14:0) and DPPC (16:0), DOPG (18:1) and CL (18:1) hold unsaturated double bonds in their longer hydrophobic tails. The length and degree of saturation of phospholipid acyl chains influence the molecular geometry of the lipids and thereby their packing in the bilayer [31], leading to rigid or fluid liposomes. Membrane fluidity is a function of the gel-to-liquid phase transition temperature T_M_ of the phospholipids, which, in turn, is dependent on the degree of saturation and directly proportional to the acyl chain length [44]. While high proportions of unsaturated and variable-length lipids in bilayers exhibit a low state of order, leading to fluid membranes, longer and saturated lipid chains lead to an increased surface for hydrophobic interactions, resulting in more rigid membranes [31]. In a physiological context (37 °C), the application of liposomes composed of DMPC and DPPC with T_M_ = 23 °C and 41 °C, respectively, implies that the vesicles would rapidly destabilize [47,48], as the phase-transition temperature of DMPC/DPPC liposomes (40%/60%) was approximated to be 34.7 °C [49]. While benefitting drug release, this behavior should hamper drug encapsulation and retention. Consistent with our observation of high storage stability of the more rigid DOPG/CL/Chol liposomes, on the other hand, phase transition is assumed to occur at higher temperatures due to the presence of cardiolipin.

### 3.2. Loading of Liposomes with **HIPS5031**

**Figure 3:**
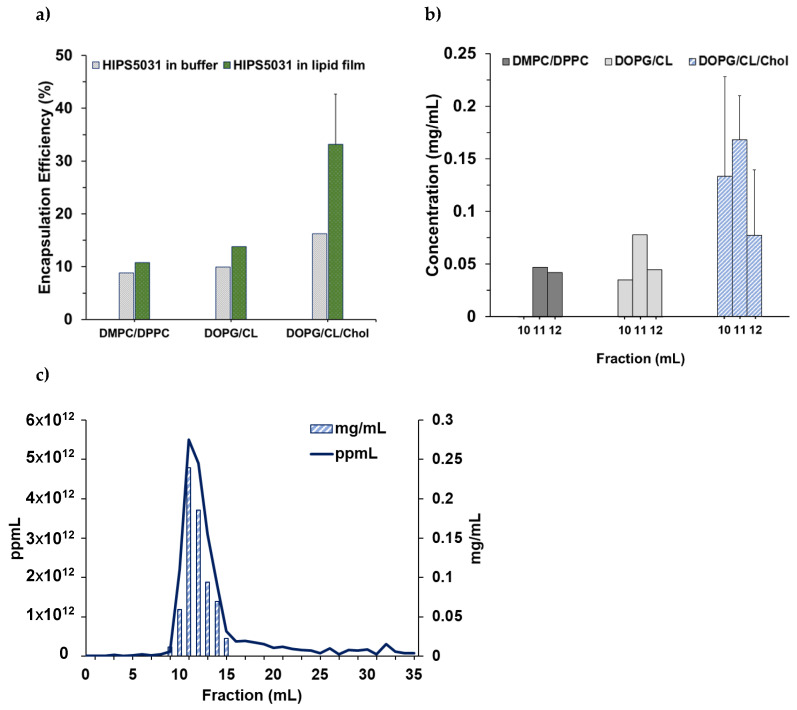
Encapsulation of **HIPS5031** into liposomes. (**a**) Encapsulation efficiency (%): bacteriomimetic and control (PC) liposomes were loaded either by rehydration of a pure lipid film with a drug-PBS solution or by rehydration of a drug-containing lipid film with PBS; (**b**) concentration of **HIPS5031** in particle-rich SEC-fractions as determined by HPLC-MS analysis (DOPG/CL/Chol *n* = 5); (**c**) SEC profile of DOPG/CL/Chol liposomes loaded with **HIPS5031**. The particle concentration of fractions was determined by NTA, and the drug concentration was determined by HPLC-MS.

Liposomes were loaded by hydration of a drug-containing lipid film, followed by extrusion and size exclusion chromatography. The encapsulation efficiency was found to be highly dependent on the solvent used for thin film preparation and on the lipid composition. Liposomes prepared from drug-containing lipid films were compared to liposomes loaded via rehydration of pure lipid films with a solution of **HIPS5031** in PBS. Dissolving the hydrophobic drug in absolute ethanol together with the lipids prior to solvent removal and thin film formation led to improved encapsulation efficiency for all lipid formulations tested (Figure 3a). This effect was particularly pronounced for the DOPG/CL/Chol liposomes. Comparison of the loading efficiency between the cholesterol-free and -enriched DOPG/CL liposomes revealed a substantial effect of the sterol component on the encapsulation of **HIPS5031**, as the EE increased 2.4-fold in the presence of cholesterol, reaching 33.2% and representing 0.17 ± 0.04 mg/mL in the SEC-fraction with the highest liposome count (Figure 3b). While the drug concentration in the liposome-rich SEC fractions and the encapsulation efficiency was the lowest for the zwitterionic DMPC/DPPC liposomes, the addition of cholesterol to the lipid composition also seemed to preserve the size and integrity of the liposomes during the loading and purification process. Nanoparticle tracking analysis of the **HIPS5031**-loaded DOPG/CL/Chol liposomes after SEC (Figure 3c) revealed particle number profiles similar to those of unloaded liposomes (data not shown). Irrespective of very high drug concentrations (HPLC-MS), the liposomes within the fraction with highest particle count retained a homogeneous size distribution with a PDI of 0.09, as measured with DLS.

**Figure 4:**
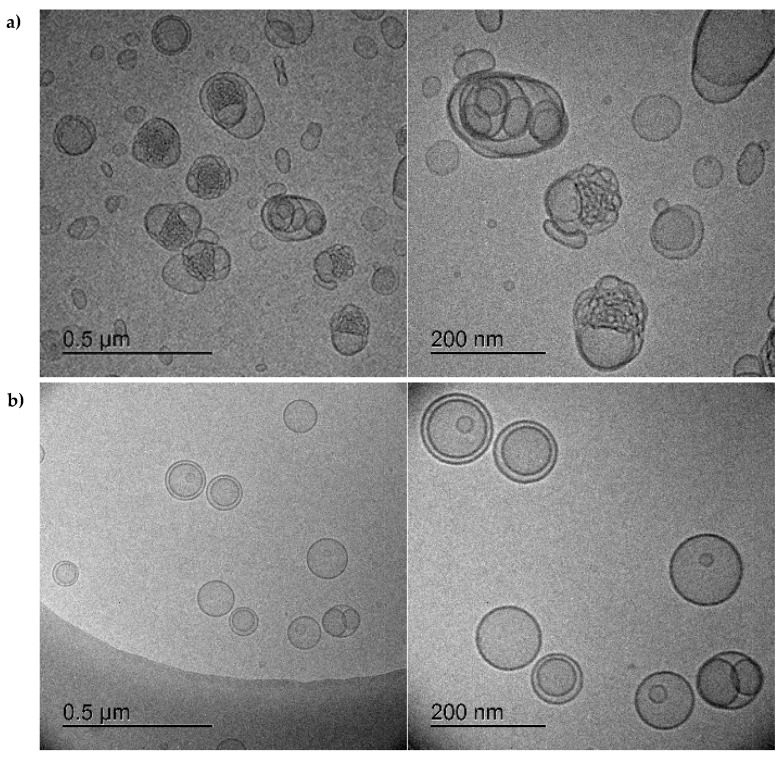
*Cryo*-TEM images of liposomes loaded with **HIPS5031**: (**a**) DOPG/CL liposomes loaded by rehydration of a pure lipid film with a drug-PBS solution. The altered morphology of vesicles indicates drug precipitation, aggregation, and vesicle fusion due to unsuccessful loading. (**b**) DOPG/CL/Chol liposomes loaded by rehydration of a drug containing dry lipid film (PBS). Oligolamellar vesicles are characterized by a smooth surface and homogenous size distribution (after SEC) without signs of instability, suggesting drug accumulation in the lipid bilayer.

The unimpaired condition of the cholesterol-enriched bacteriomimetic liposomes loaded with **HIPS5031** by rehydration of a drug-containing lipid film in comparison to the DOPG/CL liposomes loaded with a PBS-**HIPS5031** solution was further examined by *cryo*-TEM imaging (Figure 4).

The distinctively altered morphology of liposomes loaded by rehydration of **HIPS5031** dissolved in PBS (Figure 4a) could indicate unsuccessful encapsulation and instability resulting from the fusion and aggregation of vesicles with the precipitated drug. Potentially, the adsorption of drug particles onto the bilayer surface occurred instead of true encapsulation. These findings emphasize the poor aqueous solubility of **HIPS5031** and the requirement for an appropriate delivery system. When the solvent for lipid film preparation was changed from chloroform to absolute ethanol, dissolution of **HIPS5031** was possible and smooth lipid films had formed after solvent removal. Rehydrating these lipid films was found to be a very gentle and efficient strategy to successfully encapsulate the hydrophobic compound into the bilayer, as is reflected in *cryo*-TEM images revealing spherical and evenly dispersed uni- or oligolamellar vesicles with a smooth and electron-dense-appearing membrane (Figure 4b). The negligible decrease in zeta potential between the blank and loaded DOPG/CL/Chol liposomes from 43.7 ± 1.5 mV to 42.9 ± 2.6 mV indicated that improved encapsulation mainly resulted from the accumulation of **HIPS5031** in the lipophilic core of the bilayer and was unbiased by adsorption at the lipid–water interface. Beyond improving encapsulation by conveying rigidity as well as reduced permeability and drug leakage to liposomes [42,46], cholesterol might also increase the hydrophobicity of the lipophilic core and facilitate the accumulation of hydrophobic drugs when added at the optimal ratio [45].

### 3.3. ECF Transporter Assay

The effect of liposomal **HIPS5031** on vitamin uptake in prokaryotes was examined according to a recently established protocol to test the inhibitory effect of a compound against the ECF transporters [34]. This whole-cell approach allowed the study of the uptake of radiolabeled folate in a biological context using folate auxotrophic *L. casei*. While folate uptake was inhibited by 41.8 ± 10.3% in the presence of 69.66 µM of free **HIPS5031**, the liposomal formulation inhibited *L. casei* ECF transporters to an even greater extent by 65.8 ± 13.6% at the same drug concentration (Figure S3 in the SI). Interestingly, pure DOPG/CL/Chol liposomes, too, had an inhibitory effect (15.5 ± 18.9% at 2.1 × 10^11^ ppmL). This finding is suggestive of a synergistic effect of **HIPS5031** and DOPG/CL/Chol liposomes and encourages the utilization of specific lipids for liposome production.

### 3.4. Antibacterial Effect of Liposomal **HIPS5031**

**Figure 5:**
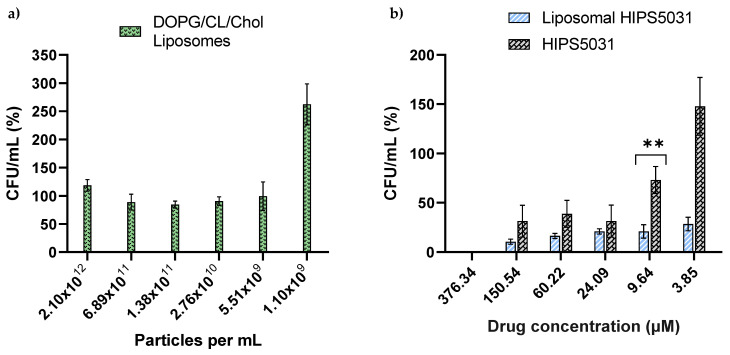
Bacterial growth inhibition assay of free vs. liposomal **HIPS5031** (DOPG/CL/Chol) performed on the Gram-positive model organism *Bacillus subtilis*. Effects were determined as a reduction in CFU/mL and were normalized to an untreated control (particle-free PBS). (**a**) Blank DOPG/CL/Chol liposomes show no intrinsic growth-inhibiting effect. The particle number (= number of liposomes) as determined by NTA was adjusted to that of drug-containing samples; in the range of 2.10 × 10^12^–5.51 × 10^9^ particles per mL, treatment with DOPG/CL/Chol liposomes did not lead to significant changes of CFU/mL compared to the control (PBS). (**b**) Liposomal **HIPS5031** reduced the growth of *B. subtilis* significantly at all concentrations tested (*p* = 0.008 at 3.85 µM) and was found to be superior to the free drug at a concentration of 9.64 µM (*p* = 0.004) (ANOVA followed by Holm–Sidak post hoc test). Values of the abscissa represent the concentration of either free or liposomal **HIPS5031**. ** *p* = 0.01.

We hypothesized beneficial drug uptake from bacteriomimetic liposomes due to their structural similarity to bacteria cell membranes [37]. Since ECF transporters are abundant in the *Firmicutes* phylum [7], **HIPS5031**-loaded DOPG/CL/Chol liposomes were tested on the model organism *B. subtilis*. To exclude intrinsic effects of DOPG/CL/Chol liposomes on the growth of *B. subtilis*, corresponding particle numbers of blank liposomes were applied as controls. The OD_600_ determination of *B. subtilis* cultures showed complete inhibition of growth after treatment with liposomal **HIPS5031** at concentrations > 57.8 ± 1.0 µM, while the free compound at the highest concentration tested (69.66 µM) only led to ~25% inhibition (a graphical representation is provided in the SI, Figure S2). In comparison, pure liposomes did not inhibit bacterial growth. In order to enumerate the antibacterial effect of **HIPS5031** and its liposomal formulation, and to validate the bacterial tolerance of pure liposomes, colony-forming units were determined. Over a range of 2.1 × 10^12^ ppmL to 5.5 × 10^9^ ppmL, representing 376.34–9.64 µM liposomal **HIPS5031**, bacterial growth was unaffected by the presence of DOPG/CL/Chol liposomes, as determined by the normalization of CFU/mL against an untreated control (PBS) (Figure 5a).

The bacterial growth inhibition assay was performed with a solution containing 376.34 µM of free or liposomal **HIPS5031** in PBS. While the growth-inhibiting effect of free **HIPS5031** subsided below a concentration of 9.64 µM (*p* = 0.035), **HIPS5031**-loaded liposomes were effective and superior to the free drug at all concentrations tested. Liposomal **HIPS5031** was effective in reducing *B. subtilis* growth up to a concentration of 9.64 µM with *p* < 0.001 and at 3.85 µM with *p* = 0.008. The superiority of the liposomal formulation over free **HIPS5031** was observed with a significant difference of a 3.5-fold reduction in CFU/mL at a concentration of 9.64 µM (*p* = 0.004) (Figure 5b).

**Figure 6:**
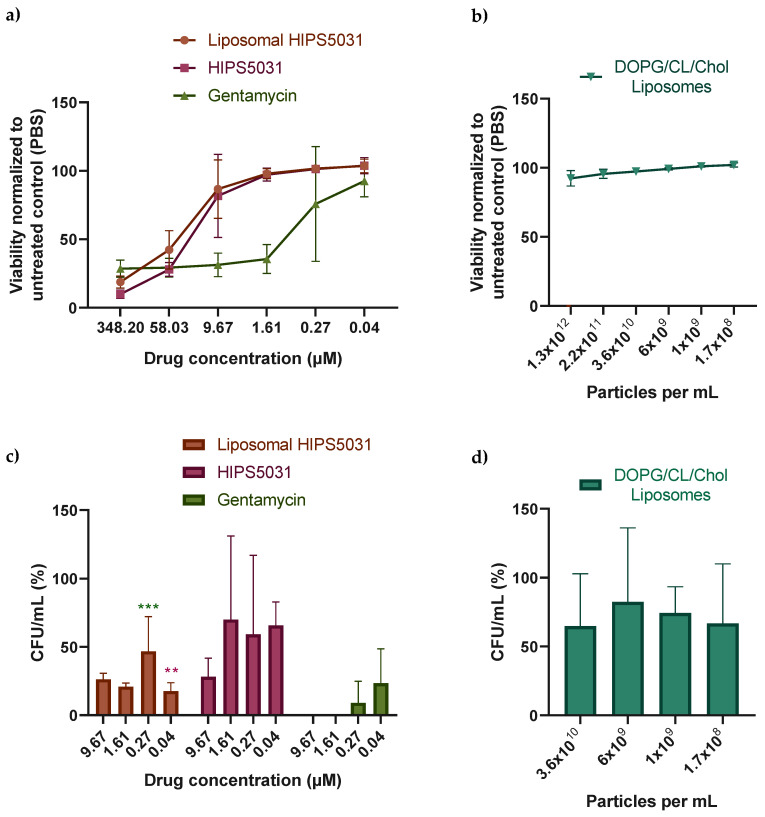
Bacterial viability assay based on the fluorescent signal derived from the reduction of resazurin to resorufin by metabolically active cells (alamarBlue HS, Invitrogen). Liquid cultures of *B. subtilis* 168 were incubated with serial dilutions of gentamicin, **HIPS5031**, and liposomal **HIPS5031** (**a**) as well as with pure liposomes (**b**). The fluorescence intensity as measured with a microplate reader (560/590 nm) was normalized to the untreated control (PBS). *n* = 9 for three biological replicates. After fluorescence measurements, colony-forming units were determined (**c**,**d**) and normalized to the untreated control (PBS). While **HIPS5031** and liposomal **HIPS5031** affected cell viability similarly and limited the regrowth of CFUs, unloaded liposomes (DOPG/CL/Chol) did not significantly impair *B. subtilis*’ viability and growth of colonies. Liposomal **HIPS5031** was less effective compared to gentamicin, but reduced CFU/mL to a larger extent than the free drug at 0.04 µM with *p* = 0.01 (multiple t-tests followed by Holm–Sidak post hoc test). *n* = 3 (biological replicates). Magenta stars = comparison to free **HIPS5031**, green stars = comparison to gentamicin. ** *p* = 0.01, *** *p* = 0.001.

As a control of our findings and in order to examine the growth-stimulating effect observed for the lowest liposome concentration (Figure 5a) and the lowest concentration of free **HIPS5031** (Figure 5b) in the CFU assay, we employed a resazurin-based viability assay (Figure 6).

*B. subtilis* cultures were incubated with solutions containing equimolar amounts of **HIPS5031**, liposomal **HIPS5031**, and gentamicin (all 348.2 µM) or with pure liposomes (DOPG/CL/Chol) and the fluorescence of resorufin, resulting from the reduction of resazurin by viable cells, was measured (Figure 6a,b). When the fluorescence intensity was compared to an untreated control (PBS), no statistically significant viability-enhancing effect of low concentrations of DOPG/CL/Chol liposomes and **HIPS5031** was observed (Figure 6c,d). While the antimicrobial effect of the ECF-transporter inhibitor **HIPS5031** was found to subside at higher concentrations compared to gentamicin, both liposomal and free **HIPS5031** effectively reduced cell viability at 348.2 µM and the loss of antimicrobial efficacy with the decreasing of the drug concentration seemed to progress similarly. Since resazurin is non-toxic to cells, CFUs were determined after the fluorescence measurement. At a concentration of 348.2 µM, no bacterial growth on Agar plates was observed (data not shown) for either gentamicin or **HIPS5031** or liposomal **HIPS5031**, suggesting a comparable antibacterial effect above the MIC. Albeit being less effective than gentamicin at low concentrations, treatment with 9.67–0.04 µM of liposomal **HIPS5031** (Figure 6c) resulted in numbers of CFU/mL that were significantly smaller compared to the untreated control (PBS). No significant difference in CFU/mL to the control (PBS) after treatment with the free drug at concentrations below 9.67 µM of free **HIPS5031** was observed. An enhanced effect of liposomal **HIPS5031** compared to free **HIPS5031**, on the other hand, was seen at a concentration of 0.04 µM, with *p* = 0.01.

These findings suggest an improved growth-inhibiting effect of liposomal **HIPS5031** compared to the free drug. We attributed this effect to the improved solubility of **HIPS5031** in DOPG/CL/Chol liposomes. Both improved solubility and sustained release from rigid, cholesterol-containing liposomes could additionally enable the formation of a concentration gradient in proximity to bacteria cells [50].

### 3.5. Anti-Biofilm Effects of Liposomal **HIPS5031**

**Figure 7:**
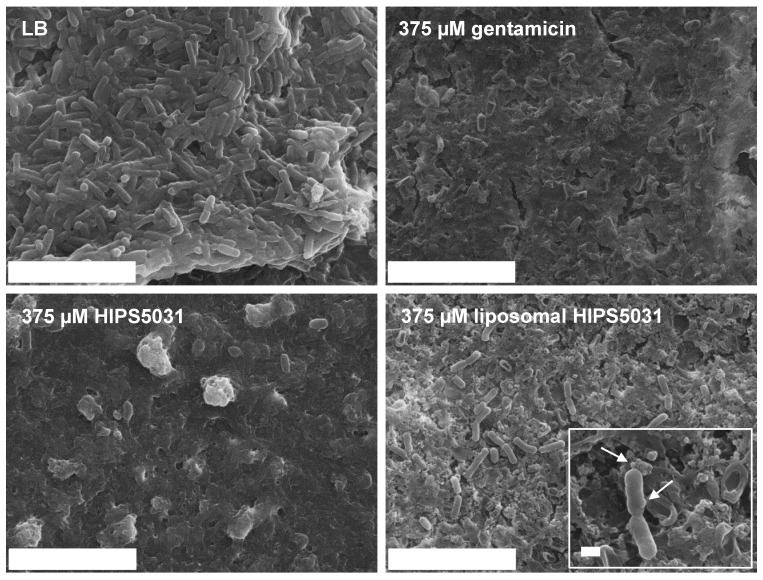
Scanning electron micrographs of *B. subtilis* 168 biofilm grown on glass coverslips in MSgg medium for 72 h prior to treatment with 375 µM of either gentamicin, **HIPS5031**, or liposomal **HIPS5031** and compared to the untreated control (LB). Scale bars = 9 µm. Inlet: arrows point at vesicular structures reminiscent of liposomes and adhering to the bacterial cell membrane; scale bar = 500 nm.

DOPG/CL/Chol liposomes display a strong negative surface charge. A previous study showed that the uptake of negatively charged and zwitterionic liposomes in eukaryotic cells is mediated by protein-dependent cellular mechanisms but does not occur via direct fusion [51]. In bacteria, moreover, the complex cell envelope poses a considerable barrier to drug permeation and shields liposomal bilayers from direct interaction with cellular membranes, as would be required for fusion or lipid exchange. The cell envelope of Gram-positive bacteria is composed of a phospholipid membrane with a superimposed peptidoglycan layer 30–100 nm in width and interspersed with proteins and anionic (lipo-) teichoic acids [52,53,54]. Permeation of antibiotics into the cytoplasm is further hampered when bacteria grow as biofilms, i.e., microbial colonies enclosed in an extracellular matrix of biopolymers (polysaccharides, (glyco-) proteins, glycolipids, and extracellular DNA) [55]. Biofilm shields the bacteria from the host immune system and leads to enhanced antibiotic recalcitrance [21,55], and hence, the efficacy of novel antibiotics against biofilm is desirable. Since nanoparticulate drug-delivery systems are able to overcome these bio-barriers [56,57], we microscopically compared the effects of DOPG/CL/Chol liposomes loaded with **HIPS5031**, free **HIPS5031**, and gentamicin against *B. subtilis* 168 biofilm. Biofilm growth of the common laboratory strain *B. subtilis* 168 was stimulated with MSgg medium [37]. Following in-lab protocols [36], biofilm for scanning electron microscopy was grown on glass coverslips for 72 h before treatment. During biofilm formation, subpopulations of bacteria alter their metabolism and morphology from short, motile rods to sessile clusters of longer cells that are encased in self-produced extracellular matrix [33]. After growth in minimal medium, this condition was observed for biofilm incubated with LB medium as a control (Figure 7). In comparison, treatment for 24 h with 375 µM each of gentamicin or free as well as liposomal **HIPS5031** led to a pronounced reduction in bacteria cells on the biofilm surface. After incubation with free **HIPS5031**, the biofilm matrix appeared unimpaired in relation to the liposomal formulation or gentamicin, the latter of which led to visible cell lysis. On the other hand, treatment with liposomal **HIPS5031** was qualitatively less effective in reducing the number of matrix-associated cells. However, spherical structures reminiscent of liposomes were visible and these vesicles seemed to interact with the bacterial membrane (arrows in inlet of Figure 7). Such a tight association between drug-loaded liposomes and cells might be of paramount importance in physiological settings.

**Figure 8:**
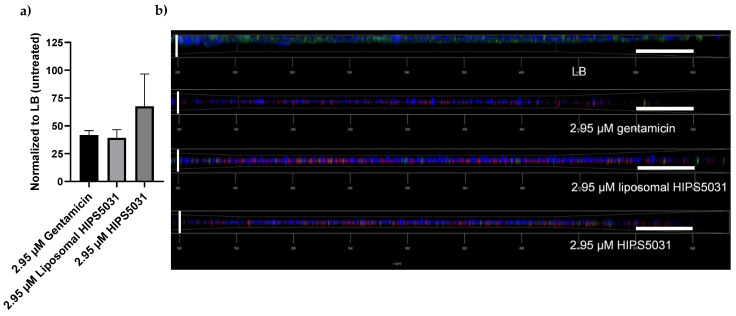
*B. subtilis* 168 biofilm for fluorescence analysis and imaging: (**a**) mean fluorescence intensity of viable bacteria cells within the preformed biofilm stained with SYTO9 after treatment with 2.95 µM of either gentamicin, **HIPS5031**, or liposomal **HIPS5031**, as measured with a microplate reader and normalized to the untreated control (LB) (*n* = 3); (**b**) confocal laser scanning micrographs of preformed biofilms treated with 2.95 µM of either gentamicin, **HIPS5031**, or liposomal **HIPS5031**, and compared to the control (LB). Biofilms were stained with Hoechst 33342, propidium iodide and SYTO9, leading to blue, green, and red fluorescence of extracellular DNA, cells, and damaged cells, respectively. Horizontal scale bars = 50 µm, vertical scale bars = 18 µm.

In order to examine the viability of cells within the biofilm after antibiotic treatment, biofilms were grown in microwell plates for 96 h and stained for confocal laser scanning microscopy [36]. Extracellular DNA is distinguished by Hoechst 33342 dye, and nucleic acid by SYTO9 and propidium iodide, the latter staining only cells with damaged membranes. Of the three fluorescent dyes employed, SYTO9-fluorescence was quantified with a microplate reader prior to confocal laser scanning microscope (CLSM) imaging. Compared to the untreated control (LB), incubation of preformed biofilms with 2.95 µM of either gentamicin or liposomal **HIPS5031** reduced the SYTO9 fluorescence intensity to an extent > 50%, while the decrease in cell viability after treatment with free **HIPS5031** did not differ significantly from either the other treatments or the control (Figure 8a). Regarding the reduction in viable cells within the biofilm, liposomal **HIPS5031** was thus assumed to have a comparable efficacy to gentamicin. On a more qualitative level but with similar implications, images obtained with the CLSM (Figure 8b) support the finding of a reduced signal of green fluorescent healthy cells after antibiotic treatment compared to the control (LB). The micrographs also showed that gentamicin and free as well as liposomal **HIPS5031** had reduced biofilm thickness, as the vertical extent of blue fluorescent extracellular DNA diminished sharply. Moreover, the intensive red fluorescence of propidium iodide emerging after antibiotic—but not LB—treatment demonstrated a clear bactericidal effect for gentamicin, but also for free and liposomal **HIPS5031**. Since both blue and red fluorescence signals were observed to be stronger after treatment with liposomal **HIPS5031** compared to the free antibiotics, it remains arguable as to whether liposomes are less effective in clearing the extracellular matrix after the killing of *B. subtilis* cells or if they exert a better bactericidal effect by enhanced penetration into mature biofilm.

Despite their qualitative and preliminary nature, these findings can be a first step in evaluating the application of ECF transporter inhibitors as new antimicrobial drugs and the characterization of liposomes as their potential delivery system. While the liposomal formulation showed a clearly improved inhibitory activity on ECF transporters and some improvement to the free drug regarding the minimal inhibitory concentration, their effects on bacterial viability and anti-biofilm activity are less unambiguous.

However, careful fine-tuning of the lipid composition and specific surface functionalization are a means by which the active uptake of drug-loaded liposomes by pathogens and even penetration through biofilm can be realized. Lipids such as phosphatidylethanolamine [23,58] or cationic DOTAP [59] are commonly employed to improve membrane fluidity or electrostatic interactions with negatively charged surface moieties of cells, respectively. These variations in lipid formulation can increase antibiotic efficacy, as was shown for the meropenem encapsulated in cationic PC/Chol/DOTAP or PC/DOPE/DOTAP liposomes, leading to 2–4 times lower MICs for *S. aureus* compared to the free drug. Cationic liposomes were superior to neutral or negatively charged liposomes [60]. Furthermore, surface decoration of nanocarriers with ligands, e.g., carbohydrates, antibodies, peptides, and proteins, allows for selective cell–receptor-mediated targeting [15,61], leading to high local concentrations and enabling penetration through biobarriers [14,38]. For example, the surface functionalization of resveratrol-loaded liposomes with cationic glyco-amphiphiles (galactosyl) was recently shown to increase the antibiotic efficacy of the quorum-sensing inhibitor against MRSA-biofilm with a 60-fold reduction in MIC compared to free resveratrol [62].

## 4. Conclusions

With this work, we showed that the encapsulation of an innovative, highly hydrophobic antibiotic lead compound into liposomes is feasible with high efficiency using an easily upscalable and accessible method. While the lipid composition is a crucial factor for drug release and liposome–cell interactions, it also determines the encapsulation efficiency. Here, the application of anionic PG and CL in combination with cholesterol was found to be beneficial for drug encapsulation compared to the cholesterol-free variant and zwitterionic PC liposomes. Improving the solubility of any drug is the principal strategy to reduce the concentrations required for successful therapy and thereby the risk of side effects. This is of particular relevance in antibiotic therapy due to the increasing resistance of bacteria to conventional treatments, often accelerated by inappropriate drug usage and dosing. The loading method for a hydrophobic new antibiotic lead compound presented in this work improved its solubility and the liposomal formulation led to an enhanced inhibition of ECF transporters and a 3.5-fold enhanced growth-inhibiting activity against *B. subtilis* when compared to the free drug at a concentration of 9.64 µM. This promising finding might be reassessed in more medically relevant pathogens demanding new therapeutic strategies. The versatility of liposomes for the encapsulation of various hydrophilic (e.g., glycopeptides [63]) and hydrophobic antibiotics (e.g., quinolones [64]), and the liposomal circumvention of generic and resistance-prone drug uptake routes into bacterial cells (e.g., porins [65]), renders liposomes a noteworthy delivery system for increasingly ineffective as well as potential new antibiotic drugs.

## Supplementary Materials

The following are available online at https://www.mdpi.com/article/10.3390/pharmaceutics14010004/s1, Figure S1: Synthetic scheme of **HIPS5031**, Figure S2: *B. subtilis* growth inhibition assay (OD_600_), Figure S3: ECF-transporter assay in *L. casei*.

## References

[1] Hutchings M.I., Truman A.W., Wilkinson B. (2019). Antibiotics: Past, present and future. Curr. Opin. Microbiol..

[2] Cassini A., Hogberg L.D., Plachouras D., Quattrocchi A., Hoxha A., Simonsen G.S., Colomb-Cotinat M., Kretzschmar M.E., Devleesschauwer B., Cecchini M. (2019). Attributable deaths and disability-adjusted life-years caused by infections with antibiotic-resistant bacteria in the EU and the European Economic Area in 2015: A population-level modelling analysis. Lancet Insfect. Dis..

[3] Antimicrobial Resistance in the EU/EEA (EARS-Net)-Annual Epidemiological Report for 2019. https://www.ecdc.europa.eu/sites/default/files/documents/surveillance-antimicrobial-resistance-Europe-2019.pdf.

[4] Chambers H.F., Deleo F.R. (2009). Waves of resistance: Staphylococcus aureus in the antibiotic era. Nat. Rev. Microbiol..

[5] Tacconelli E., Carrara E., Savoldi A., Harbarth S., Mendelson M., Monnet D.L., Pulcini C., Kahlmeter G., Kluytmans J., Carmeli Y. (2018). Discovery, research, and development of new antibiotics: The WHO priority list of antibiotic-resistant bacteria and tuberculosis. Lancet Infect. Dis..

[6] Bousis S., Setyawati I., Diamanti E., Slotboom D.J., Hirsch A.K.H. (2019). Energy-Coupling Factor Transporters as Novel Antimicrobial Targets. Adv. Ther..

[7] Slotboom D.J. (2014). Structural and mechanistic insights into prokaryotic energy-coupling factor transporters. Nat. Rev. Microbiol..

[8] Bao Z., Qi X., Hong S., Xu K., He F., Zhang M., Chen J., Chao D., Zhao W., Li D. (2017). Structure and mechanism of a group-I cobalt energy coupling factor transporter. Cell Res..

[9] Dokoumetzidis A., Macheras P. (2006). A century of dissolution research: From Noyes and Whitney to the biopharmaceutics classification system. Int. J. Pharm..

[10] Day T., Read A.F. (2016). Does High-Dose Antimicrobial Chemotherapy Prevent the Evolution of Resistance?. PLoS Comput. Biol..

[11] Vasseur M.V., Laurentie M., Rolland J.G., Perrin-Guyomard A., Henri J., Ferran A.A., Toutain P.L., Bousquet-Melou A. (2014). Low or high doses of cefquinome targeting low or high bacterial inocula cure Klebsiella pneumoniae lung infections but differentially impact the levels of antibiotic resistance in fecal flora. Antimicrob. Agents Chemother..

[12] Diamanti E., Setyawati I., Bousis S., Souza P.C.T., Mojas L., Swier L., Haupenthal J., Gibson P., Volz C., Stanek W. (2021). Targeting the energy-coupling factor (ECF) transporters: Identification of new tool compounds. ChemRXiv.

[13] Kalepu S., Nekkanti V. (2015). Insoluble drug delivery strategies: Review of recent advances and business prospects. Acta Pharm. Sin. B.

[14] Gonzalez Gomez A., Hosseinidoust Z. (2020). Liposomes for Antibiotic Encapsulation and Delivery. ACS Infect. Dis..

[15] Ferreira M., Ogren M., Dias J.N.R., Silva M., Gil S., Tavares L., Aires-Da-Silva F., Gaspar M.M., Aguiar S.I. (2021). Liposomes as Antibiotic Delivery Systems: A Promising Nanotechnological Strategy against Antimicrobial Resistance. Molecules.

[16] Lee Y., Thompson D.H. (2017). Stimuli-responsive liposomes for drug delivery. Wiley Interdiscip. Rev. Nanomed. Nanobiotechnol..

[17] Su F.-Y., Chen J., Son H.-N., Kelly A.M., Convertine A.J., West T.E., Skerrett S.J., Ratner D.M., Stayton P.S. (2018). Polymer-augmented liposomes enhancing antibiotic delivery against intracellular infections. Biomater. Sci..

[18] Goes A., Fuhrmann G. (2018). Biogenic and Biomimetic Carriers as Versatile Transporters To Treat Infections. ACS Infect. Dis..

[19] Drulis-Kawa Z., Dorotkiewicz-Jach A. (2010). Liposomes as delivery systems for antibiotics. Int. J. Pharm..

[20] Khameneh B., Diab R., Ghazvini K., Fazly Bazzaz B.S. (2016). Breakthroughs in bacterial resistance mechanisms and the potential ways to combat them. Microb. Pathog..

[21] Rukavina Z., Vanić Ž. (2016). Current Trends in Development of Liposomes for Targeting Bacterial Biofilms. Pharmaceutics.

[22] Alhariri M., Azghani A., Omri A. (2013). Liposomal antibiotics for the treatment of infectious diseases. Expert Opin. Drug Deliv..

[23] Diab R., Khameneh B., Joubert O., Duval R. (2015). Insights in Nanoparticle-Bacterium Interactions: New Frontiers to Bypass Bacterial Resistance to Antibiotics. Curr. Pharm. Des..

[24] Epand R.F., Savage P.B., Epand R.M. (2007). Bacterial lipid composition and the antimicrobial efficacy of cationic steroid compounds (Ceragenins). Biochim. Biophys. Acta.

[25] Sohlenkamp C., Geiger O. (2016). Bacterial membrane lipids: Diversity in structures and pathways. FEMS Microbiol. Rev..

[26] Khalifat N., Fournier J.B., Angelova M.I., Puff N. (2011). Lipid packing variations induced by pH in cardiolipin-containing bilayers: The driving force for the cristae-like shape instability. Biochim. Biophys. Acta.

[27] Lewis R.N., McElhaney R.N. (2009). The physicochemical properties of cardiolipin bilayers and cardiolipin-containing lipid membranes. Biochim. Biophys. Acta.

[28] Tomšiè N., Babnik B., Lombardo D., MavčIč B., Kandušer M., Iglič A., Kralj-Iglič V. (2005). Shape and Size of Giant Unilamellar Phospholipid Vesicles Containing Cardiolipin. J. Chem. Inf. Modeling.

[29] Clejan S., Krulwich T.A., Mondrus K.R., Seto-Young D. (1986). Membrane lipid composition of obligately and facultatively alkalophilic strains of Bacillus spp.. J. Bacteriol..

[30] Goes A., Lapuhs P., Kuhn T., Schulz E., Richter R., Panter F., Dahlem C., Koch M., Garcia R., Kiemer A.K. (2020). Myxobacteria-Derived Outer Membrane Vesicles: Potential Applicability Against Intracellular Infections. Cells.

[31] Belin B.J., Busset N., Giraud E., Molinaro A., Silipo A., Newman D.K. (2018). Hopanoid lipids: From membranes to plant–bacteria interactions. Nat. Rev. Microbiol..

[32] Gallegos-Monterrosa R., Mhatre E., Kovács Á.T. (2016). Specific Bacillus subtilis 168 variants form biofilms on nutrient-rich medium. Microbiology.

[33] Vlamakis H., Chai Y., Beauregard P., Losick R., Kolter R. (2013). Sticking together: Building a biofilm the Bacillus subtilis way. Nat. Rev. Microbiol..

[34] Bousis S., Winkler S., Haupenthal J., Fulco F., Diamanti E., Hirsch A.K.H. (2021). An efficient way to screen inhibitors of energy-coupling factor (ECF) transporters in bacteria uptake assay. ChemRXiv.

[35] Wiegand I., Hilpert K., Hancock R.E.W. (2008). Agar and broth dilution methods to determine the minimal inhibitory concentration (MIC) of antimicrobial substances. Nat. Protoc..

[36] Goes A., Vidakovic L., Drescher K., Fuhrmann G. (2021). Interaction of myxobacteria-derived outer membrane vesicles with biofilms: Antiadhesive and antibacterial effects. Nanoscale.

[37] Branda S.S., Gonzalez-Pastor J.E., Ben-Yehuda S., Losick R., Kolter R. (2001). Fruiting body formation by Bacillus subtilis. Proc. Natl. Acad. Sci. USA.

[38] Wang D.Y., van der Mei H.C., Ren Y., Busscher H.J., Shi L. (2019). Lipid-Based Antimicrobial Delivery-Systems for the Treatment of Bacterial Infections. Front. Chem..

[39] Berbel Manaia E., Paiva Abuçafy M., Chiari-Andréo B.G., Lallo Silva B., Oshiro-Júnior J.A., Chiavacci L. (2017). Physicochemical characterization of drug nanocarriers. Int. J. Nanomed..

[40] Fan Y., Marioli M., Zhang K. (2021). Analytical characterization of liposomes and other lipid nanoparticles for drug delivery. J. Pharm. Biomed. Anal..

[41] Danaei M., Dehghankhold M., Ataei S., Hasanzadeh Davarani F., Javanmard R., Dokhani A., Khorasani S., Mozafari M. (2018). Impact of Particle Size and Polydispersity Index on the Clinical Applications of Lipidic Nanocarrier Systems. Pharmaceutics.

[42] Anderson M., Omri A. (2004). The Effect of Different Lipid Components on the In Vitro Stability and Release Kinetics of Liposome Formulations. Drug Deliv..

[43] Duplessis J., Ramachandran C., Weiner N., Muller D. (1996). The influence of lipid composition and lamellarity of liposomes on the physical stability of liposomes upon storage. Int. J. Pharm..

[44] Rideau E., Dimova R., Schwille P., Wurm F.R., Landfester K. (2018). Liposomes and polymersomes: A comparative review towards cell mimicking. Chem. Soc. Rev..

[45] Briuglia M.-L., Rotella C., McFarlane A., Lamprou D.A. (2015). Influence of cholesterol on liposome stability and on in vitro drug release. Drug Deliv. Transl. Res..

[46] Raffy S., Teissié J. (1999). Control of lipid membrane stability by cholesterol content. Biophys. J..

[47] Gregoriadis G. (1991). Overview of liposomes. J. Antimicrob. Chemother..

[48] Pietzyk B., Henschke K. (2000). Degradation of phosphatidylcholine in liposomes containing carboplatin in dependence on composition and storage conditions. Int. J. Pharm..

[49] Buffo F., Sierra M., Pedroni V., Morini M. (2017). Lipidic nanoparticules: A model function to predict the transition temperature of DPPC-DMPC mixtures. Adv. Mater. Sci..

[50] Furneri P.M., Fresta M., Puglisi G., Tempera G. (2000). Ofloxacin-Loaded Liposomes: In Vitro Activity and Drug Accumulation in Bacteria. Antimicrob. Agents Chemother..

[51] Montizaan D., Yang K., Reker-Smit C., Salvati A. (2020). Comparison of the uptake mechanisms of zwitterionic and negatively charged liposomes by HeLa cells. Nanomedicine.

[52] Bharatiya B., Wang G., Rogers S.E., Pedersen J.S., Mann S., Briscoe W.H. (2021). Mixed liposomes containing gram-positive bacteria lipids: Lipoteichoic acid (LTA) induced structural changes. Colloids Surf. B Biointerfaces.

[53] Desvaux M.L., Dumas E., Chafsey I., Hebraud M. (2006). Protein cell surface display in Gram-positive bacteria: From single protein to macromolecular protein structure. FEMS Microbiol. Lett..

[54] Silhavy T.J., Kahne D., Walker S. (2010). The Bacterial Cell Envelope. Cold Spring Harb. Perspect. Biol..

[55] Flemming H.-C., Wingender J. (2010). The biofilm matrix. Nat. Rev. Microbiol..

[56] Gao W., Thamphiwatana S., Angsantikul P., Zhang L. (2014). Nanoparticle approaches against bacterial infections. Wiley Interdiscip. Rev. Nanomed. Nanobiotechnol..

[57] Kirtane A.R., Verma M., Karandikar P., Furin J., Langer R., Traverso G. (2021). Nanotechnology approaches for global infectious diseases. Nat. Nanotechnol..

[58] Kent B., Garvey C.J., Cookson D., Bryant G. (2009). The inverse hexagonal-inverse ribbon-lamellar gel phase transition sequence in low hydration DOPC:DOPE phospholipid mixtures. Chem. Phys. Lipids.

[59] Smith M.C., Crist R.M., Clogston J.D., McNeil S.E. (2017). Zeta potential: A case study of cationic, anionic, and neutral liposomes. Anal. Bioanal. Chem..

[60] Drulis-Kawa Z., Gubernator J., Dorotkiewicz-Jach A., Doroszkiewicz W., Kozubek A. (2006). A comparison of the in vitro antimicrobial activity of liposomes containing meropenem and gentamicin. Cell. Mol. Biol. Lett..

[61] Mitchell M.J., Billingsley M.M., Haley R.M., Wechsler M.E., Peppas N.A., Langer R. (2021). Engineering precision nanoparticles for drug delivery. Nat. Rev. Drug Discov..

[62] Aiello S., Pagano L., Ceccacci F., Simonis B., Sennato S., Bugli F., Mancini G. (2021). Mannosyl, glucosyl or galactosyl liposomes to improve resveratrol efficacy against Methicillin Resistant Staphylococcus aureus biofilm. Colloids Surf. A: Physicochem. Eng. Asp..

[63] Sande L., Sanchez M., Montes J., Wolf A.J., Morgan M.A., Omri A., Liu G.Y. (2012). Liposomal encapsulation of vancomycin improves killing of methicillin-resistant Staphylococcus aureus in a murine infection model. J. Antimicrob. Chemother..

[64] Ribeiro L.N.D.M., De Paula E., Rossi D.A., Monteiro G.P., Júnior E.C.V., Silva R.R., Franco R.R., Espíndola F.S., Goulart L.R., Fonseca B.B. (2020). Hybrid Pectin-Liposome Formulation against Multi-Resistant Bacterial Strains. Pharmaceutics.

[65] Makabenta J.M.V., Nabawy A., Li C.-H., Schmidt-Malan S., Patel R., Rotello V.M. (2021). Nanomaterial-based therapeutics for antibi-otic-resistant bacterial infections. Nat. Rev. Microbiol..

