# Supplementary material for: Bacteriomimetic Liposomes Improve Antibiotic Activity of a Novel Energy-Coupling Factor Transporter Inhibitor
Source: Pharmaceutics. 2021 Dec 21;14(1):4. doi: 10.3390/pharmaceutics14010004 (PMC8779172; doi:10.3390/pharmaceutics14010004)
Supplement: Supplementary file 1 [file pharmaceutics-14-00004-s001.zip › pharmaceutics-1443339-supplementary.pdf]

Supplementary Information

Bacteriomimetic liposomes improve antibiotic activity of a novel energy-coupling factor transporter inhibitor

Menka Drost[1,6], Eleonora Diamanti[2,4], Kathrin Fuhrmann[1], Adriely Goes[1], Atanaz Shams[2], Jörg Haupenthal[2], Marcus Koch[3], Anna KH Hirsch[2,4,5] and Gregor Fuhrmann[1,6]*

menka.drost@fau.de; gregor.fuhrmann@fau.de

1 Helmholtz Centre for Infection Research (HZI), Biogenic Nanotherapeutics Group (BION), Helmholtz Institute for Pharmaceutical Research Saarland (HIPS), Campus E8.1, 66123 Saarbrücken, Germany

2 Helmholtz Centre for Infection Research (HZI), Department of Drug Design an Optimization (DDOP), Helmholtz-Institute for Pharmaceutical Research Saarland (HIPS), Campus E8.1, Saarbrücken, 66123, Germany

3 INM - Leibniz-Institut für Neue Materialien, Campus D2.2, Saarbrücken, 66123, Germany

4 Helmholtz International Lab for Anti-Infectives, Campus E8.1, 66123 Saarbrücken, Germany

5 Department of Pharmacy, Saarland University, Campus C1.7, Saarbrücken, 66123, Germany

6 Friedrich-Alexander-University Erlangen-Nürnberg (FAU), Department of Biology, Pharmaceutical Biology, Staudtstr. 5, 91058 Erlangen

* Correspondence: gregor.fuhrmann@fau.de

Table of content

1. HIPS5031
   1.1. General information on chemicals, materials and methods
   1.2. Synthetic scheme
   1.3. Characterization of HIPS5031
2. Bacterial growth assay (*Bacillus subtilis ssp. subtilis*)
3. ECF transporter assay

1. HIPS5031

1.1. General information on chemicals, materials and methods

HIPS5031 is a derivative of an ECF transporter inhibitor described by Diamanti et al. [1]. NMR experiments were run on a Bruker Ultrashield plus 500 (500 MHz) spectrometer. Spectra were acquired at 300 K, using deuterated dimethylsulfoxide (DMSO-d6) as solvent. Chemical shifts for 1H and 13C spectra were recorded in parts per million (ppm) using the residual non-deuterated solvent as the internal standard (for DMSO d6: 2.50 ppm, 1H; 39.52 ppm, 13C). Coupling constants (J) are given in Hertz (Hz). Data are reported as follows: chemical shift, multiplicity (s = singlet, d =doublet, t = triplet, m = multiplet, br = broad and combinations of these) coupling constants and integration. Flash chromatography was performed using the automated flash chromatography system CombiFlash Rf+ (Teledyne Isco, Lincoln, NE, USA) equipped with RediSepRf silica columns (Axel Semrau, Sprockhövel Germany). TLC was performed with aluminium-backed silica TLC plates (Macherey-Nagel MN ALUGRAM Sheets SIL G/UV 254 20 x 20cm 818133) with a suitable solvent system and was visualized using UV fluorescence (254 & 366 nm). All reactions were carried out in oven-dried glassware under an atmosphere of argon. Anhydrous DMF was purchased from Aldrich and used directly.

Mass spectrometry was performed on a SpectraSystems-MSQ LCMS system (Thermo Fisher, Dreieich, Germany) using a Hypersil Gold column, 150 x 3 mm, 5 µm. At a flow rate of 700 µL/min, the gradient of H_2O (0.1% FA) and ACN (0.1% FA) starting from 30% ACN and then increased to 95% over 12 min. The mass spectrum was measured in positive and negative mode in a range from 100–600 m/z. The UV spectrum was recorded at 254 nm. High-resolution mass spectra (HR-MS) were recorded with a ThermoScientific system where a Dionex Ultimate 3000 RSLC was coupled to a Q Exactive Focus mass spectrometer with an electrospray ion (ESI) source. An Acquity UPLC® BEH C8, 150 x 2.1 mm, 1.7 µm column equipped with a VanGuard Pre-Column BEH C8, 5 x 2.1 mm, 1.7 µm (Waters, Germany) was used for separation. At a flow rate of 250 µL/min, the gradient of (A) H_2O + 0.1% FA and (B) ACN + 0.1% FA was held at 10% B for 1 min and then increased to 95% B over 4 min. It was held there for 1.2 min before the gradient was decreased to 10% B over 0.3 min where it was held for 1 min. The mass spectrum was measured in positive mode in a range from 120–1000 m/z. UV spectrum was recorded at 254 nm.

Abbreviations dimethylformamide (DMF), dimethylsulfoxide (DMSO), ethyl acetate (EtOAc), hydrochloric acid (HCl), sodium sulfate (Na2SO4), sodium hydride 60% dispersion in mineral oil (NaH 60%).Other abbreviations used are: hours (h), room temperature (rt), on (overnight).

1.2. Synthetic scheme

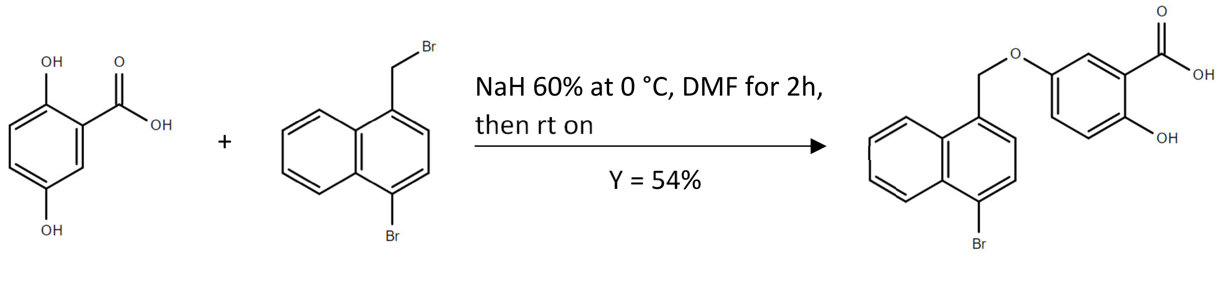

Figure S1. Synthesis of **HIPS5031** (5-((4-Bromonaphthalen-1-yl)methoxy)-2-hydroxybenzoic acid).

General procedure:

To a stirred solution of 5-hydroxy-2-substituted benzoic acid (0.200 g, 1.3 mmol) in DMF (4.3 mL, 0.3 M), was added at 0 °C portionwise NaH (60%, 2.5 eq) previously washed with diethyl ether (3x5 mL). The reaction mixture was stirred at rt for 2 h, and then 1-bromo-4-(bromomethyl)naphthalene (0.390 g, 1.3 mmol) was slowly added at 0 °C. The reaction mixture was stirred at rt on until TLC and LC-MS analysis showed the complete consumption of the starting materials. The resulting suspension was quenched at 0 °C with HCl (6 N) and extracted with EtOAc (3x10 mL). The combined organic layers were dried over Na_2SO_4, filtered, concentrated *in vacuo* and purified by flash chromatography (hexane/EtOAc 1:9) to give **HIPS5031** as white powder (0.260 g, 54%).

1.3. Characterization of **HIPS5031**

^{1}H NMR (500 MHz, DMSO) δ 8.20 (m, 2H), 7.90 (d, *J* = 7.6, 1H), 7.72 (m, 2H), 7.59 (d, *J* = 7.7, 1H), 7.45 (d, *J* = 3.2, 1H), 7.28 (dd, *J* = 9.0, 3.2, 1H), 6.92 (d, *J* = 9.0, 1H), 5.52 (s, 2H).

^{13}C NMR (126 MHz, DMSO d_6) δ 171.5, 155.6, 150.4, 133.1, 132.3, 131.2, 129.5, 127.8, 127.4, 127.2, 126.9, 124.8, 124.2, 122.3, 118.1, 114.2, 112.7, 68.0.

HRMS (ESI) calcd for $C_{18}H_{12}BrO_4$ $(M\text{-}H)^-$: 370.99972, found: 370.99210.

2. Bacterial growth assay (*Bacillus subtilis ssp. subtilis*)

Experiments to determine the growth of strain *B. subtilis ssp. subtilis* were performed as previously described [2]. Percent growth inhibitions were determined at different concentrations of inhibitor and liposomes. These were added to the bacteria either individually or in combination, at concentrations ranging from 6.65×10^{9} to 2.1×10^{11} ppmL (liposomes) and 2.18 to 69.66 µM (compound). ODs at 600 nm were determined after a final time point (16 h after addition of inhibitor and/or liposomes) in a CLARIOstar Platereader (BMG Labtech, Ortenberg, Germany), followed by a graphical representation (Figure S2).

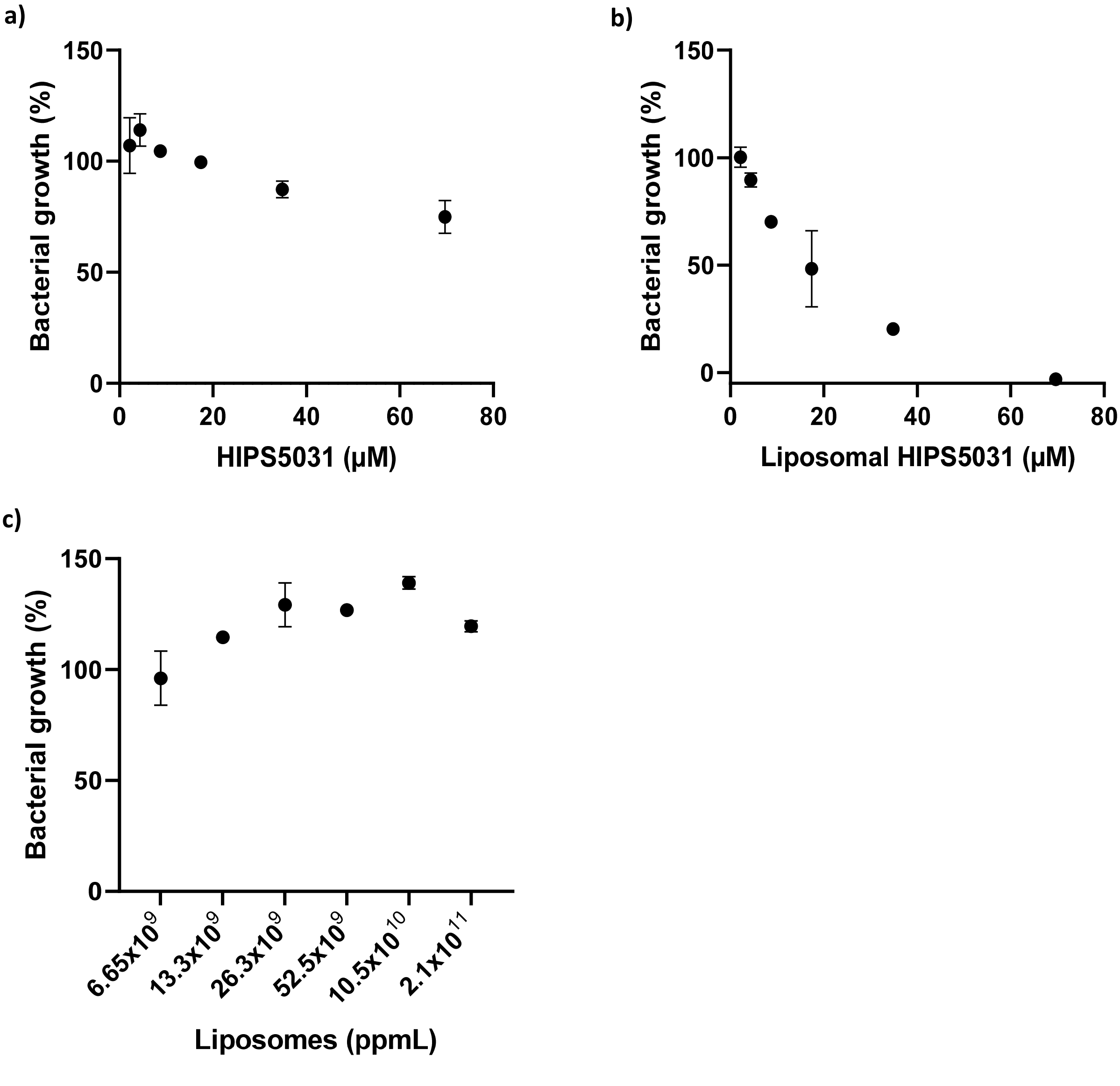

Figure S2. Effect of HIPS5031, liposomal HIPS5031 (DOPG/CL/Chol) or pure liposomes (DOPG/CL/Chol) on *B. subtilis* growth as determined by OD_{600}. Minimal inhibitory concentration of liposomal HIPS5031 = 57.8 ± 1.0 µM. ppmL = particles per mL. Data are presented as mean ± SD of two independent experiments.

3. ECF transporter assay

The inhibitory effect of free and liposomal HIPS5031 on ECF-transporter mediated folate uptake in the Gram-positive model organism *L. casei* was studied in a cell-based transport assay as described recently [3], with slight modifications. 20 µL of either pure liposomes, free HIPS5031, or liposomal HIPS5031 was added to the wells of a MultiScreen HTS Filter Plate - GV (Merck KGaA, Darmstadt, Germany) containing 175 µL of a *L. casei* culture diluted in citrate buffer. Stock solutions of free HIPS5031 contained 2% DMSO. DOPG/CL/Chol liposomes, free HIPS5031 or liposomal HIPS5031 were each tested at three different concentrations and compared to 175 µL of the bacterial suspension + 20 µL of PBS as negative control. The blank was determined with 185 µL citrate buffer + 10 µL of DMSO. 5 µL of radiolabeled folic acid was added to each well (2 µM, Moravek Biochemicals, Brea, CA).

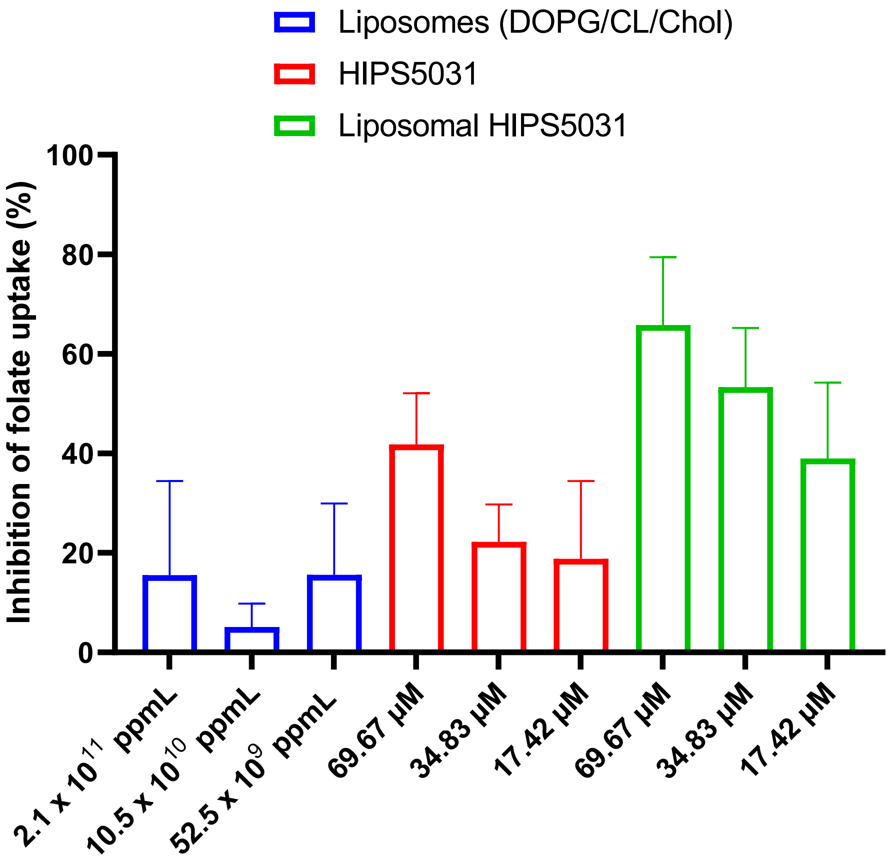

Figure S3. Inhibition of folate uptake in *L. casei*. Uptake of radiolabeled folate was assessed in a whole-cell based assay. Liposomes were composed of DOPG/CL/Chol. The liposome concentration of pure liposomes (particles per mL, ppmL) is corresponding to the liposome concentration of loaded liposomes (liposomal HIPS5031). Data are presented as mean ± SD of 4 independent experiments.

References

1. Diamanti, E.; Setyawati, I.; Bousis, S.; Souza, P.C.T.; Mojas, L.; Swier, L.; Haupenthal, J.; Gibson, P.; Volz, C.; Stanek, W.; et al. Targeting the energy-coupling factor (ECF) transporters: identification of new tool compounds. **2021**, doi:10.26434/chemrxiv-2021-xq08b-v2.
2. Elgaher, W.A.M.; Fruth, M.; Groh, M.; Haupenthal, J.; Hartmann, R.W. Expanding the scaffold for bacterial RNA polymerase inhibitors: design, synthesis and structure–activity relationships of ureido-heterocyclic-carboxylic acids. *RSC Adv.* **2014**, *4*, 2177-2194, doi:10.1039/c3ra45820b.
3. Bousis, S.; Winkler, S.; Haupenthal, J.; Fulco, F.; Diamanti, E.; Hirsch, A.K.H. An efficient way to screen inhibitors of energy-coupling factor (ECF) transporters in bacteria uptake assay. **2021**, doi:10.26434/chemrxiv-2021-b50gj.
